# Longitudinal analysis of regional brain changes in anti-NMDAR encephalitis: a case report

**DOI:** 10.1186/s12883-021-02446-8

**Published:** 2021-10-27

**Authors:** Ryan M. Nillo, Iris J. Broce, Besim Uzgil, Nilika S. Singhal, Christine M. Glastonbury, Christopher P. Hess, James A. Barkovich, Rahul S. Desikan, Leo P. Sugrue

**Affiliations:** 1 UCSF, Department of Radiology and Biomedical Imaging, Neuroradiology Section, 513 Parnassus Avenue, S-255, San Francisco, CA 94143 USA; 2 UCSF, Department of Neurology, San Francisco, CA USA

**Keywords:** Encephalitis, Volumetric MRI, Autoimmune disease, Seizures, Longitudinal analysis

## Abstract

**Background:**

Anti-NMDA receptor encephalitis is an immune-mediated disorder characterized by antibodies against the GluN1 subunit of the NMDA receptor that is increasingly recognized as a treatable cause of childhood epileptic encephalopathy. In adults, the disorder has been associated with reversible changes in brain volume over the course of treatment and recovery, but in children, little is known about its time course and associated imaging manifestations.

**Case presentation:**

A previously healthy 20-month-old boy presented with first-time unprovoked seizures, dysautonomia, and dyskinesia. Paraneoplastic workup was negative, but CSF was positive for anti-NMDAR antibodies. The patient’s clinical condition waxed and waned over a 14-month course of treatment with first- and second-line immunotherapies (including steroids, IVIG, rituximab, and cyclophosphamide). Serial brain MRIs scans obtained at 5 time points spanning this same period showed no abnormal signal or enhancement but were remarkable for cycles of reversible regional cortical volume loss. All scans included identical 1-mm resolution 3D T1-weighted sequences obtained on the same 3 T scanner. Using a novel longitudinal processing stream in FreeSurfer6 (Reuter M, et. al, Neuroimage 61:1402–18, 2012) we quantified the rate of change in cortical volume at each vertex (% volume change per month) between consecutive scans and correlated these changes with the time course of the patient’s treatment and clinical response. We found regionally specific changes in cortical volume (up to 7% per month) that preferentially affected the frontal and occipital lobes and paralleled the patient’s clinical course, with clinical decline associated with volume loss and clinical improvement associated with volume gain.

**Conclusions:**

Our results suggest that reversible cortical volume loss in anti-NMDA encephalitis has a regional specificity that mirrors many of the clinical symptoms associated with the disorder and tracks the dynamics of disease severity over time. This case illustrates how quantitative morphometric techniques can be applied to clinical imaging data to reveal patterns of brain change that may provide insight into disease pathophysiology. More widespread application of this approach might reveal regional and temporal patterns specific to different types of autoimmune encephalitis, providing a tool for diagnosis and a surrogate marker for monitoring treatment response.

## Background

Anti-N-methyl-D-aspartate receptor (anti-NMDAR) encephalitis is an immune-mediated disorder characterized by antibodies against the GluN1 subunit of the NMDA receptor (reviewed in [1]). Frequently paraneoplastic, and first described in women with ovarian teratomas, in adults the disorder often presents with neuropsychiatric symptoms and has been associated with reversible changes in brain volume over the course of treatment and recovery [2]. In children, it is increasingly recognized as a treatable cause of encephalitis, with younger children often presenting with seizures, dystonia, or mutism [3]. Here we use quantitative morphometric methods to characterize longitudinal changes in brain volume in a 20-month-old boy with anti-NMDAR encephalitis and related autoimmune epileptic encephalopathy over a 14-month period, showing cycles of reversible regional cortical volume loss that preferentially affect the frontal and occipital lobes and parallel the patient’s treatment and clinical course.

## Methods

### Data acquisition and analysis

With institutional review board approval, the patient’s imaging and electronic medical record were reviewed. This review did not require consent, however, written informed consent was obtained from the patient’s parents prior to publication. 3D T1-weighted brain MRI at 1.0 mm effective isotropic resolution was acquired on a 3 T scanner (Discovery 750, GE Healthcare) at 5 time points: initial presentation, and follow-up scans at 3, 9, 11, and 14 months. Scans employed very similar sequence parameters spanning the following ranges: TR: 10–11 msec; TE: 4.2–4.7 msec; Flip Angle: 15 degrees; matrix: 512–512, sagittal acquisition with 150–166 slices. Using longitudinal processing in FreeSurfer6 [4], we quantified the rate of change in volume at each vertex on the cortical surface between consecutive time points ((volume_2_ – volume_1_)/(time_2_-time_1_) × 1/volume_1_)). This approach first creates an unbiased subject-specific template across all available time points and then uses common information from this template in subsequent processing steps to increase the reliability and statistical power of longitudinal analyses. Our regions of interest (frontal, parietal, temporal, occipital, and ‘cingulate’ lobes) were created by parcellating the brain according to the Desikan-Killiany atlas [5], grouping these regions into lobes, and finally averaging lobes between hemispheres. The initial cortical surface reconstructions and segmentations were performed using a validated, automated surface-based cortical segmentation method included in the FreeSurfer6 software package [5]. Results are expressed as percent change in volume per month between consecutive time points with respect to the earlier time point.

## Case presentation

### Case description and qualitative imaging results

**Fig. 1:**
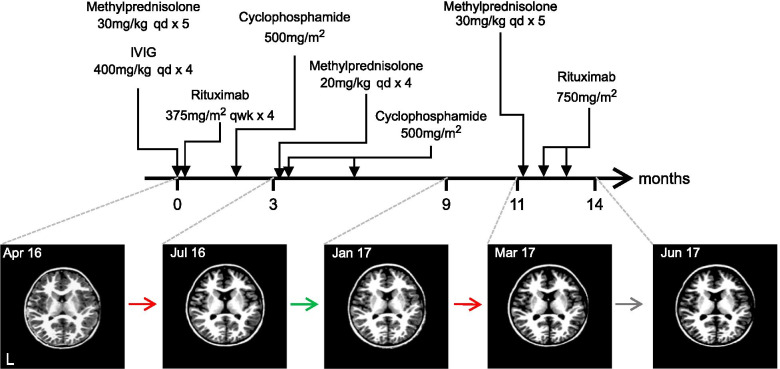
Timeline of treatments and imaging. Timeline shows relative timing of treatments received (top) with respect to imaging performed (bottom) at 0, 3, 9, 11 and 14 months after presentation. Representative axial T1 images from each scan are shown at the same approximate anatomic level (L indicates the left hemisphere). Colored arrows summarize the overall change in volume between scans (as quantified in Fig. 2) with red, green, and grey signifying losses, gains, and no change respectively

In April 2016, a previously healthy 20-month-old boy presented with first-time unprovoked seizure and abnormal movements. Figure 1 shows the timeline of the patient’s subsequent treatment and brain imaging. His initial neurological exam was notable for irritability, dysautonomia, choreoathetosis, and severe oro-facial dyskinesias. Brain MRI at presentation was normal but CSF showed an inflammatory pleocytosis and positive anti-NMDAR antibodies. Paraneoplastic workup including whole-body MRI, PET-CT, and two scrotal ultrasounds, was negative for malignancy. The patient did not improve after standard first-line therapy for NMDAR encephalitis (30 mg/kg IV methylprednisolone for 5 days, followed by IVIG 2 g/kg) prompting further treatment with second-line immunotherapies: First, 4 cycles of IV rituximab (375 mg/m^2^/week) and, when recovery plateaued, IV cyclophosphamide (500 mg/m^2^).

A second MRI obtained in mid-July 2016, 3-months after initial presentation, showed extensive cortical volume loss. At that time, worsening seizure burden, disorganized background EEG, and persistently abnormal CSF inflammatory markers prompted a repeat course of steroids (20 mg/kg IV methylprednisolone, qd x 4d) and cyclophosphamide (500 mg/m^2^). The patient was finally discharged in late July 2016, and over the next several months motor control, developmental milestones, and seizures all improved.

An MRI in January 2017, 9-months after initial presentation, showed marked recovery of cortical volume. Shortly after this scan, however, the patient experienced clinical decline with neurodevelopmental regression and increased frequency of multiple seizure types unresponsive to anti-epileptic medications. A fourth brain MRI in March 2017 showed new cortical volume loss compared to January. This presumed progression of autoimmune epileptic encephalopathy was again treated with high-dose steroids and a course of rituximab with dramatic improvement in inter-ictal EEG and resolution of seizures. Over subsequent months, the patient made neurodevelopmental progress in speech, ambulation, and manual dexterity. Despite this clinical improvement, a fifth and final brain MRI performed in June 2017, showed minimal interval change compared to the March 2017 study.

### Quantifying longitudinal cortical change

**Fig. 2:**
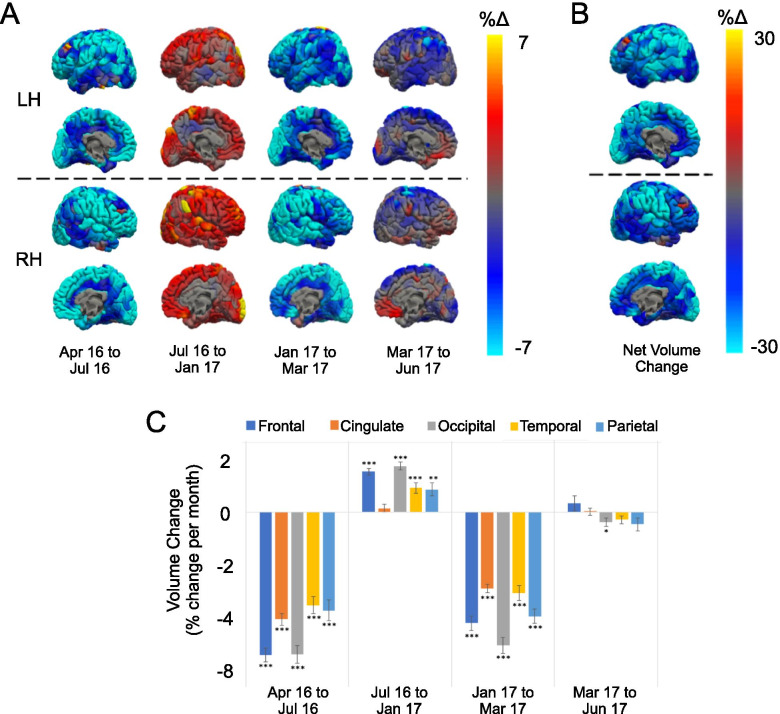
Regional quantification of longitudinal changes in cortical volume. **A**: 3D renderings show areas of cortical volume increases (red to yellow) and decreases (blue to cyan) as percent volume change per month between consecutive scans based on a vertex-wide analysis. Changes between consecutive scans were mapped to a temporal average and smoothed at FWHM = 15 mm. **B**: Net volume change across a 14-month period. (C): Bar graphs show average percentage change per month in lobar volume between consecutive scans. Asterisks highlight changes that are significantly different from zero (* *p* ≤ 0.05, ** *p* ≤ 0.01 and *** *p* < =0.001)

Between successive time points, we found significant regionally-specific changes in cortical volume. These included reversible volume losses and gains that were greatest in the frontal and occipital lobes and smallest in the temporal lobes (Fig. 2A & C). Over a period of several months, these changes tracked the patient’s clinical status and treatment (Fig. 1), with periods of clinical decline (Apr 16 to Jul 16 and Jan 17 to Mar 17) preceding volume loss and periods of clinical improvement (Jul 16 to Jan 17) preceding volume gain. Ultimately, these fluctuations converged on a pattern of stable net cortical volume loss, again most prominent in the frontal and occipital lobes (Fig. 2B).

## Conclusion

We used quantitative longitudinal analysis to show that anti-NMDAR encephalitis in a pediatric patient preferentially affected the frontal and occipital lobes and paralleled the patient’s clinical course – with volume loss associated with clinical worsening and volume gain with periods of clinical recovery. To our knowledge, this is the youngest patient described with NMDAR encephalitis and related autoimmune epileptic encephalopathy.

NMDAR encephalitis is thought to result from antibody-mediated internalization of NMDA receptors [1], with selective involvement of inhibitory neurons hypothesized to account for increased cortical excitability and seizures. During the active phase of the disease, our data suggest that affected brain regions recover following immunosuppressive therapy, manifest as increased cortical volume and improved epileptic encephalopathy. Recovery of brain volume after anti-NMDAR encephalitis has been reported as a qualitative phenomenon in adult patients who were rescanned years after treatment [2]. Here we quantify the regional specificity and the dynamics of brain volume changes over the course of treatment.

Two potential confounds might affect our conclusions. First, our patient received three courses of corticosteroids, which have been associated with reversible short-term decreases in brain volume [6]. The cause and timing of steroid-related brain volume changes remains unclear, however, loss of intracellular water has been proposed as a mechanism [7] and in patients with multiple-sclerosis, volume loss following short-term steroid treatment resolved within 30–60 days [8]. In the case of our patient timing argues against a simple steroid effect as the first scan –showing volume loss after initial presentation (July 16)– occurred 3 months following steroid treatment, and the second scan –showing volume loss in the context of new symptoms following initial clinical and radiographic recovery (March 17)– was not preceded by any interval steroid treatment. Second, our patient experienced a waxing and waning complex seizure disorder consistent with epileptic encephalopathy. Specifically, he experienced cognitive and behavioral regression and mixed seizure types including tonic seizures with EEG demonstrating runs of slow spike-wave discharges in the background, as well as periods of generalized paroxysmal fast activity (GPFA). Taken together, these clinical and electrographic findings demonstrate the development of an epileptic encephalopathy, best characterized as the Lennox-Gastaut Syndrome (LGS). An association between LGS and cerebral volume loss has been described [9], so it is possible that the volume changes we observed could reflect manifestations of seizures rather than the underlying encephalopathy. However, a growing literature suggests that autoimmunity is an under-recognized cause of ‘idiopathic’ pediatric seizure disorders [10]. Thus, while we cannot prove the ultimate cause of cerebral volume changes in this patient, the clinical and imaging history strongly suggest reversible volume loss associated with a steroid-responsive autoimmune encephalopathy due to anti-NMDAR antibodies. Indeed, at our institution, we now routinely screen for autoimmune causes in all cases of pediatric epileptic encephalopathy and infantile spasms.

Intriguingly, the regional pattern of cortical volume changes in this patient, with the largest changes in the frontal and occipital lobes, mirrors many of the clinical symptoms associated with anti-NMDAR encephalitis. Behavioral and psychiatric symptoms, reflecting frontal lobe dysfunction, are among the most well-established symptoms, however, Probasco et al. [11] recently proposed visual impairment to be an under-recognized but specific clinical feature of anti-NMDAR encephalitis. Specifically, these researchers reported medial occipital lobe hypometabolism identified by FDG-PET/CT in patients with acute anti-NMDAR encephalitis and visual symptoms. Interestingly, patients with de novo mutations in *GRIN1,* the gene encoding the GluN1 subunit of the NMDA receptor, experience a chronic encephalopathy that also includes a combination of motor, cognitive, and visual symptoms [12], suggesting that a specific clinical phenotype may result from dysfunction in the GluN1 NMDA receptor subunit, whether genetic or acquired. It is interesting to speculate whether the reversible pattern of volume changes observed in our patient reflects an imaging marker of this phenotype.

Finally, this case illustrates how quantitative longitudinal morphometric techniques applied to clinical data can reveal patterns of brain change that may provide insight into underlying disease pathophysiology. More widespread application of this approach might reveal distinct regional and temporal patterns in different types of autoimmune encephalitis, providing a tool for diagnosis and a surrogate marker for treatment response.

## Data Availability

Anonymized imaging data (skull stripped T1-weighted scans for each time point) will be shared by the corresponding author on reasonable request from any qualified investigator.

## Abbreviations

NMDAR: N-methyl-D-aspartate receptor
